# A meta-analysis of public microarray data identifies biological regulatory networks in Parkinson’s disease

**DOI:** 10.1186/s12920-018-0357-7

**Published:** 2018-04-13

**Authors:** Lining Su, Chunjie Wang, Chenqing Zheng, Huiping Wei, Xiaoqing Song

**Affiliations:** 1 Department of Biology of Basic Medical Science College, Hebei North University, Zhangjiakou, 075000 Hebei China; 2 Department of Basic Medicine, Zhangjiakou University, Zhangjiakou, 75000 Hebei China; 3 Shenzhen RealOmics (Biotech) Co., Ltd, Shenzhen, 518081 Guangdong China

**Keywords:** Parkinson’s disease, Long non-coding RNA, microRNA, SNPs, Network

## Abstract

**Background:**

Parkinson’s disease (PD) is a long-term degenerative disease that is caused by environmental and genetic factors. The networks of genes and their regulators that control the progression and development of PD require further elucidation.

**Methods:**

We examine common differentially expressed genes (DEGs) from several PD blood and substantia nigra (SN) microarray datasets by meta-analysis. Further we screen the PD-specific genes from common DEGs using GCBI. Next, we used a series of bioinformatics software to analyze the miRNAs, lncRNAs and SNPs associated with the common PD-specific genes, and then identify the mTF-miRNA-gene-gTF network.

**Result:**

Our results identified 36 common DEGs in PD blood studies and 17 common DEGs in PD SN studies, and five of the genes were previously known to be associated with PD. Further study of the regulatory miRNAs associated with the common PD-specific genes revealed 14 PD-specific miRNAs in our study. Analysis of the mTF-miRNA-gene-gTF network about PD-specific genes revealed two feed-forward loops: one involving the SPRK2 gene, hsa-miR-19a-3p and SPI1, and the second involving the SPRK2 gene, hsa-miR-17-3p and SPI. The long non-coding RNA (lncRNA)-mediated regulatory network identified lncRNAs associated with PD-specific genes and PD-specific miRNAs. Moreover, single nucleotide polymorphism (SNP) analysis of the PD-specific genes identified two significant SNPs, and SNP analysis of the neurodegenerative disease-specific genes identified seven significant SNPs. Most of these SNPs are present in the 3′-untranslated region of genes and are controlled by several miRNAs.

**Conclusion:**

Our study identified a total of 53 common DEGs in PD patients compared with healthy controls in blood and brain datasets and five of these genes were previously linked with PD. Regulatory network analysis identified PD-specific miRNAs, associated long non-coding RNA and feed-forward loops, which contribute to our understanding of the mechanisms underlying PD. The SNPs identified in our study can determine whether a genetic variant is associated with PD. Overall, these findings will help guide our study of the complex molecular mechanism of PD.

**Electronic supplementary material:**

The online version of this article (10.1186/s12920-018-0357-7) contains supplementary material, which is available to authorized users.

## Background

Parkinson’s disease (PD) is one of the most common neurodegenerative diseases. The main symptoms of PD include shaking, bradypragia and postural instability [1]. PD is characterized by the loss of dopaminergic neurons in the substantia nigra (SN) in brain, which involves the increase of microglia, and the presence of Lewy bodies [2].

Many studies have indicated that PD may be the result of a combination of genetic and environmental factors, such as exposure to pesticides, metals, solvents, and other toxicants [3]. Approximately 15% of individuals affected by PD have a family member with the disease [4] and 5–10% of people with PD are affected by a single specific gene mutation [5]. Previous studies have indicated that mutations in several specific genes cause PD, including genes encoding alpha-synuclein (SNCA), leucine-rich repeat kinase 2 (LRRK2), glucocerebrosidase (GBA), parkin (PRKN), PTEN-induced putative kinase 1 (PINK1), parkinson disease protein 7 (PARK7), vacuolar protein sorting-associated protein 35 (VPS35), eukaryotic translation initiation factor 4 gamma 1 (EIF4G1), dnaJ heat shock protein family (Hsp40) member C13 (DNAJC13) and coiled-coil-helix-coiled-coil-helix domain containing 2 (CHCHD2) [6]. The SNCA gene plays an important role in PD, because the encoded protein is the main component of Lewy bodies, which accumulate in the brains of people with PD [7]. Mutations in the SNCA gene have been found in different groups with sporadic (non-familial) PD and familial PD [5]. Mutations in the LRRK2 gene, which encodes a protein called dardarin, are associated with many familial and sporadic PD patients [8]. A recent study showed that carriers of the G2385R variant in the LRRK2 gene showed more of a tendency towards fatigue than non-carriers in PD patients [9]. A mutation in GBA is proposed to be the greatest genetic risk of PD through its effects in increasing the levels of SNCA [10]. The GBA variant E326K (rs2230288) was significantly more frequent in PD patients compared with controls, indicating that this variant is a susceptibility allele for PD [10]. Mutations in PINK1, PRKN, and PARK7 genes may cause mitochondrial dysfunction, which are observed in PD [11]. Some evidence has shown that low concentrations of urate in the blood serum increase the risk of PD [12].

These above studies improve our understanding of the molecular mechanism in PD. However, discordance among those studie make the combination of results from many similar studies difficult. However, meta-analysis uses statistical techniques to get combining information from multiple existing studies can increase the power and generalizability of results in PD [13]. A limited number of meta studies has been conducted on gene expression profiles to identify novel biomarkers for PD. However, different microarray data combination, different sizes of each individual study combination, and different statistical methods are among the factors contributed to the different results. For instance, Elisa Mariani et al. [14] used TRAM Software to conduct a meta-analysis of PD transcriptome data. Chang D et al. [15] carried out a meta-analysis with a recent study of over 13,000 PD cases and 95,000 controls with estimating the h2 value for PD, and then identified 17 new PD risk loci. Wang Q et al. [16] performed a meta-analysis with 9 microarray datasets of PD studies in every brain region using the RankProd method [17].

Our meta-analysis was conducted on PD patients and healthy controls microarray datasets obtained from the SN brain region and blood using Vote counting generic ways. In this study, five independent SN microarrays (GSE7621, GSE8397-GPL96, GSE8397-GPL97, GSE20163, and GSE20141) from PD patients and healthy controls were integrated and analyzed to screen common differentially expressed genes (DEGs). We also performed a meta-analysis of three independent blood microarrays (GSE99039, GSE6613, and GSE72267) from PD patients and healthy controls to screen common DEGs.

MicroRNAs (miRNAs) are small non-coding RNA molecules (~ 22 nt long) that are found in plants, animals and some viruses and have been proven to play crucial roles in gene expression. Long non-coding RNAs (lncRNAs), longer than 200 nt, can control gene expression by interacting with the miRNA pathways [18]. LncRNAs binds to miRNAs through miRNA response elements or binding sites to regulate miRNA target gene expression [18]. Several studies have revealed that non-coding RNAs, such as miRNAs and lncRNAs, have been implicated in PD pathogenesis [13].

Finally, we examined the regulatory network involving genes, miRNAs, transcription factors (TFs) and lncRNA in PD progression to better understand the molecular mechanisms involved in this disease. In our study, we use some same bioinformatics software and similar methodological workflow as the recently published paper [13].

## Methods

**Fig. 1:**
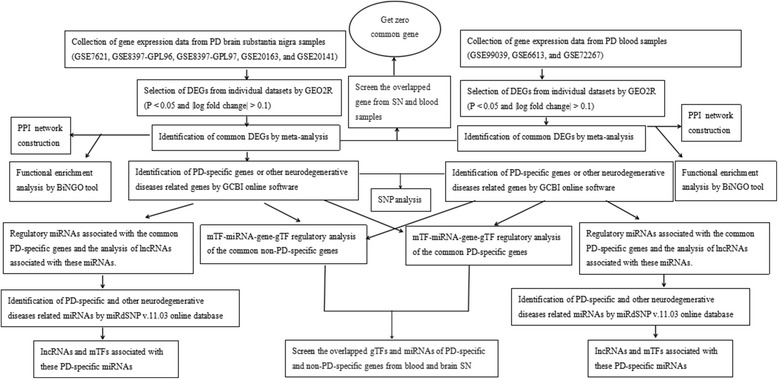
Workflow of the methodology used in our study

Our overarching goal was to identify candidate biomarkers of PD (the focus of this study) using the follow workflow (Fig. 1).

### Data collection

We used “Parkinson’s disease” as keywords to search for genome-wide expression studies in NCBI-GEO (http://www.ncbi.nlm.nih.gov/geo/) and EMBL-EBI ArrayExpress databases (https://www.ebi.ac.uk/arrayexpress/). Only original experimental studies that screened for different genes between PD and healthy humans were the first choice for inclusion. Additional inclusion criteria included the following: (1) the study type was expression profiling by array; (2) Studies which comprised of cell intensity file (CEL) raw files were available; and (3) studies about blood or brain SN in PD were used in the analysis. With the inclusion criteria, three datasets from blood and five datasets from SN in PD were screened. We then performed meta-analysis of three datasets from blood studies (GSE99039, GSE6613, and GSE72267) and five datasets from SN studies (GSE7621, GSE8397-GPL96, GSE8397-GPL97, GSE20163, and GSE20141). Details of the microarray datasets are provided in Table 1.

**Table 1:** Datasets used in the meta-analysis

Tissue	GEO Accession	Sample Size(control/PD)	Platform	PMID
Blood	GSE99039	PD = 205; HC = 233	GPL570: Affymetrix Human Genome U133 Plus 2.0 Array	28,916,538 [90]
	GSE6613	PD = 50; HC = 22	GPL96: Affymetrix Human Genome U133A Array	17,215,369 [91]
	GSE72267	PD = 40; HC = 19	GPL571: Affymetrix Human Genome U133A 2.0 Array	26,510,930 [92]
SN	GSE7621	PD = 16; HC = 9	GPL570: Affymetrix Human Genome U133 Plus 2.0 Array	17,571,925 [93]
	GSE8397-GPL96	PD = 24; HC = 15	GPL96: Affymetrix Human Genome U133A Array;	16,344,956 [94]
	GSE8397-GPL97	PD = 24; HC = 15	GPL97: Affymetrix Human Genome U133B Array	
	GSE20141	PD = 10; HC = 8	GPL570: Affymetrix Human Genome U133 Plus 2.0 Array	20,926,834 [95]
	GSE20163	PD = 8; HC = 9	GPL96: Affymetrix Human Genome U133A Array	20,926,834 [95]

*PD* Parkinson’s disease, *HC* healthy control

Searches were executed up to August 2017.

### Inclusion criteria for DEGs

GEO2R is an interactive web tool that is based on GEOquery and limma R packages from the Bioconductor project that is used to identify DEGs by comparing different groups of GEO Series samples [19]. In this study, each individual dataset was processed using the GEO2R online software to compare healthy control samples and PD patient samples to screen DEGs. The t test and Benjamini and Hochberg method were used to calculate the *P* values and FDR, respectively [20]. Genes that were differentially expressed between PD and healthy controls were screened with the threshold value of *P* < 0.05 and |log fold change| > 0.1. The probes with no gene annotation or matched multiple gene symbols were removed, and when multiple probes matched to the same gene symbol, we selected the probe with the highest |log fold change| value.

### Meta-analysis of DEGs of gene expression microarray datasets

Here, Meta-analysis was performed on the three datasets from blood studies and five datasets from brain SN independently using Vote counting generic ways of combining information [21]. The results were visualized using a Venn diagram that were plotted using the OmicShare tools, a free online platform for data analysis (http://www.omicshare.com/tools).

Next, we mainly used GCBI online software to identify common DEGs associated with PD as PD-specific genes, and to identify other neurodegenerative diseases as other neurodegenerative diseases related genes.

### Functional enrichment analysis

BiNGO is a tool to determine the Gene Ontology (GO) categories that are statistically overrepresented in a set of genes or a subgraph of a biological network [22]. BiNGO was used to analyze GO enrichment of DEGs in blood tissues and in brain SN of PD. GO enrichment with *p* < 0.05 and false discovery rate (FDR) < 0.05 was regarded as statistically significant.

### Protein-protein interaction (PPI) network construction for common DEGs

The selected common genes from SN tissue and blood were subjected to STRING v.10.5 database [23] analysis to construct PPI networks. The threshold value was a score of 0.4. PPI networks were visualized by Cytoscape software v. 3.4.0 [24] and analyzed using the Network Analyzer tool based on degree. The degree indicates the number of interactions of a particular protein. The size of the node is proportional to the degree in the interaction network. The more the nodes connected to the node, the larger the node will be. Large nodes indicate bigger degrees. In this network, we selected some genes that are with a high node degree. At last, the top three nodes with degree values above the average network degree value were identified.

### Biological regulatory interaction and networks for common PD-specific genes from our study

Experimentally validated miRNA and common PD-specific gene interactions were analyzed using DIANA-Tarbase [25]. We also examined lncRNA and miRNA interactions using DIANA-LncBase Experimental v.2 [26]. This database contains more than 70,000 low and high-throughput, (in)direct miRNA: lncRNA experimentally supported interactions, derived from manually curated publications and the analysis of 153 AGO CLIP-Seq libraries [26]. Experimentally validated (prediction score ≥ 0.90) lncRNAs were selected in our study.

To study molecular regulator interactions, we built a regulatory network comprising common PD-specific genes, TFs associated with the genes, miRNAs associated with the genes and TFs associated with the miRNAs. Information on TFs binding site that were associated with the genes was obtained from TRANSFAC [27] based on the Match™ algorithm. The TRANSFAC database contains data from a wide variety of eukaryotic organisms, ranging from human to yeast and comprising data on transcription factors, their target genes and regulatory binding sites [27]. In our study, the search algorithm uses two score values, the matrix similarity score (MSS) and the core similarity score (CSS), to estimate the result. Finally, the inclusion criteria was score = 1 for the MSS and CSS. The gene-miRNA interaction information was analyzed using DIANA-Tarbase [25]. Regulatory information on the TFs associated with these miRNAs was obtained from the TransmiR database [28]. We then generated the network using Cytoscape software v. 3.4.0 [24].

PD-specific and other neurodegenerative diseases related miRNAs identified from miRNAs associated with the PD-specific genes were filtered from miRdSNP v.11.03 online database [29].

### The analysis of lncRNAs and mTFs associated with these PD-specific miRNAs related to the PD-specific genes

To identify possible lncRNA-mediated regulation of the PD-specific miRNAs associated with common PD-specific genes of our study, we collected regulatory information in the lncbase module of DIANA-LncBase v.2 [26]. The experimentally validated lncRNAs (score ≥ 0.90) were selected in our study. Regulatory information on the TFs that were associated with these miRNAs was obtained from the TransmiR v.1.2 database [26]. We then got a regulatory pattern involving the TFs, lncRNAs and common PD-specific genes associated with the PD-specific miRNAs.

### Regulatory analysis of the common non-PD-specific genes

To study the molecular regulator interactions with the common non-PD-specific genes, we perform regulatory analysis including TFs associated with the genes, miRNAs associated with the genes and TFs associated with the miRNAs. Information on the TFs that were associated with the genes was obtained from TRANSFAC [27] and the inclusion criteria was score = 1for the MSS and CSS. The gene-miRNA interaction information was analyzed using DIANA-Tarbase v.7.0 [25], miRWalk database v.3.0 [20] and TargetScan human v.7.1 [30]. Regulatory information on the TFs that were associated with these miRNAs was obtained from the TransmiR v.1.2 database [28].

PD-specific miRNAs identified from miRNAs associated with the common non-PD-specific genes were filtered from miRdSNP v.11.03 online database [29].

### SNP analysis of the common PD-specific and other neurodegenerative disease-specific genes

To identify PD-associated SNPs, the common genes in our study were subjected to SNP analysis. SNPs corresponding to these genes were identified from the MirSNP online database (http://bioinfo.bjmu.edu.cn/mirsnp/search/) [31]. We obtained a large number of SNPs related to the genes. To identify the PD-specific or neurodegenerative disease-specific SNPs from this large number of SNPs, we screened the SNPs in miRdSNP v.11.03 [29] and LincSNP v.2.0 [32]. Chromosome locus and allele gene information corresponding to each of the SNPs were searched using the dbSNP database(https://www.ncbi.nlm.nih.gov/snp/?term=).

## Results

### Meta-analysis of DEGs and PD-specific or other neurodegenerative diseases related DEGs identification

**Fig. 2:**
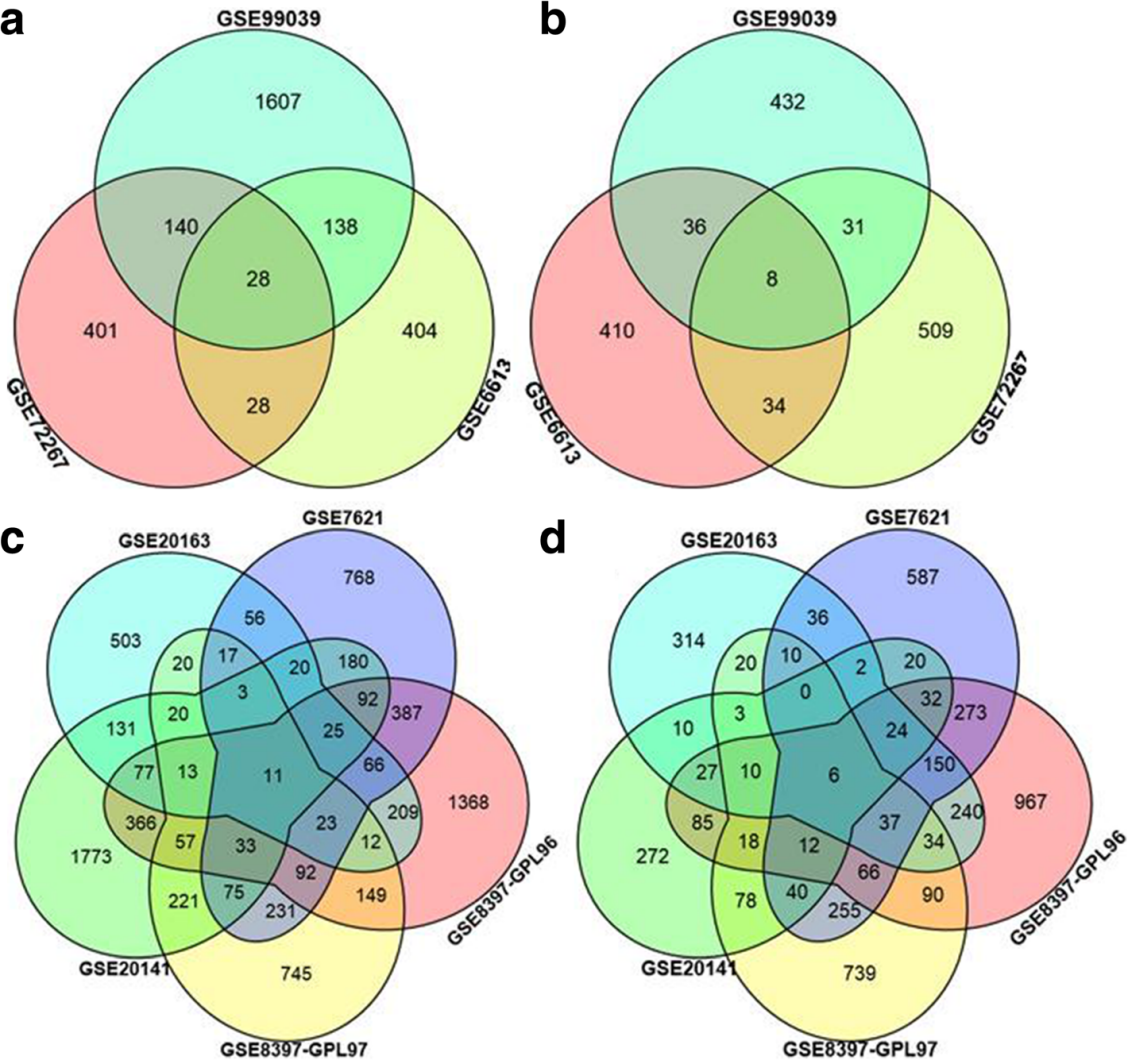
The number of common genes obtained from blood and substantia nigra (SN) expression profiling datasets visualized by a Venn diagram. **a** The number of downregulated genes in expression profiling datasets from blood; **b** The number of upregulated genes in expression profiling datasets from blood; **c** The number of downregulated genes in expression profiling datasets from SN; and **d** The number of upregulated genes in expression profiling datasets from SN

To identify DEGs between PD and healthy controls, we used three datasets from blood and five datasets from the brain SN region to perform meta-analyses and detected common genes across these datasets. The analyses revealed that 28 downregulated genes and 8 upregulated genes were differentially expressed in the three blood studies (Fig. 2a, b and Table 2). Out of the total 36 DEGs in the blood studies, 19 were previously known to be associated with several other neurodegenerative diseases, such as Alzheimer’s disease and four common genes (HSPA6, MAP2K6, SRPK2 and NOL7) were also previously associated with PD (named PD-specific genes)(identified using GCBI online software). We also identified 11 downregulated and 6 upregulated genes as differentially expressed in the five brain SN studies (Fig. 2c, d and Table 3). Among the 17 total DEGs in the brain SN studies, nine genes have been previously associated with neurodegenerative diseases, and one gene (SNCA) was identified as PD-specific genes(identified using GCBI online software). Overall, the number of downregulated genes was greater than the number of upregulated genes.

**Table 2:** Common differentially expressed genes identified from blood in PD patients and healthy controls and that PD-specific and other neurodegenerative diseases related genes identification

Expression	Gene symbol	Gene name	Corresponding neurodegenerative disease other than PD
Down-regulated	ABCA1^a^	ATP binding cassette subfamily A member 1	Alzheimer’s disease
	ABHD5	abhydrolase domain containing 5	
	ADGRG3	adhesion G protein-coupled receptor G3	
	AKAP13^a^	A-kinase anchoring protein 13	Alzheimer’s disease
	APMAP^a^	adipocyte plasma membrane associated protein	Multiple sclerosis; Alzheimer’s disease
	ARG1	arginase 1	
	BAZ1A^a^	bromodomain adjacent to zinc finger domain 1A	Huntington disease
	BMX^a^	BMX non-receptor tyrosine kinase	Alzheimer’s disease
	CDKL5^a^	cyclin dependent kinase like 5	
	CEBPD^a^	CCAAT/enhancer binding protein delta	Alzheimer’s disease
	CSF2RA^a^	colony stimulating factor 2 receptor alpha subunit	Alzheimer’s disease
	CTBP2^a^	C-terminal binding protein 2	Alzheimer’s disease
	FAM120A^a^	family with sequence similarity 120A	Multiple sclerosis
	HLA-C^a^	major histocompatibility complex, class I, C	Multiple sclerosis
	HSPA6^b^	heat shock protein family A (Hsp70) member 6	
	IRS2	insulin receptor substrate 2	
	LILRB1	leukocyte immunoglobulin like receptor B1	
	LRRFIP1^a^	LRR binding FLII interacting protein 1	Multiple sclerosis; Huntington disease
	MAP2K6^b^	mitogen-activated protein kinase kinase 6	
	MAP2K7	mitogen-activated protein kinase kinase 7	
	MGAM	maltase-glucoamylase	
	NCAM1	neural cell adhesion molecule 1	
	NCOA3	nuclear receptor coactivator 3	
	PML^a^	promyelocytic leukemia	Polyglutamine diseases
	SRPK2^b^	SRSF protein kinase 2	
	SUPT20H^a^	SPT20 homolog, SAGA complex component	Cerebellar Purkinje cell degeneration
	THOC2^a^	THO complex 2	Multiple sclerosis
	TMX4^a^	thioredoxin related transmembrane protein 4	Motor neuron disease
Up-regulated	ATM	ATM serine/threonine kinase	
	BCL2^a^	BCL2, apoptosis regulator	Alzheimer’s disease
	FAM102A^a^	family with sequence similarity 102 member A	Neurodegenerative disease
	LRRN3	leucine rich repeat neuronal 3	
	NOL7^b^	nucleolar protein 7	
	TCF3	transcription factor 3	
	TP73-AS1	TP73 antisense RNA 1	
	YME1L1^a^	YME1 like 1 ATPase	Alzheimer’s disease

^a^Previously associated with several neurodegenerative diseases (identified using GCBI online software), but not PD

^b^Previously associated with PD (identified using GCBI online software)

**Table 3:** Common differentially expressed genes identified from substantia nigra in PD patients and healthy controls, and PD-specific and other neurodegenerative diseases related genes identification

Expression	Gene symbol	Gene name	Corresponding neurodegenerative disease other than PD
Down-regulated	CARHSP1^a^	calcium regulated heat stable protein 1	Multiple sclerosis
	GART^a^	phosphoribosylglycinamide formyltransferase phosphoribosylglycinamide synthetase, phosphoribosylaminoimidazole synthetase	Alzheimer’s disease
	MUC4	mucin 4, cell surface associated	
	NIN	ninein	
	NRF1^a^	nuclear respiratory factor 1	Alzheimer’s disease
	PRELP^a^	proline and arginine rich end leucine rich repeat protein	Nervous system disease; Neurodegenerative disease
	RGS12	regulator of G-protein signaling 12	
	RNF130^a^	ring finger protein 130	Motor neuron disease
	SNAP23^a^	synaptosome associated protein 23	Nervous system disease
	SNTB2	syntrophin beta 2	
	TBX1	T-box 1	
Up-regulated	ACSL6^a^	acyl-CoA synthetase long-chain family member 6	Nervous system disease
	ATP5S^a^	ATP synthase, H+ transporting, mitochondrial Fo complex subunit s (factor B)	Alzheimer’s disease
	CADPS	calcium dependent secretion activator	
	DCLK1^a^	doublecortin like kinase 1	Alzheimer’s disease
	PPFIA2	PTPRF interacting protein alpha 2	
	SNCA^b^	synuclein alpha	

^a^Previously associated with several neurodegenerative diseases, other than PD (identified using GCBI online software)

^b^Previously associated with PD (PD-specific) (identified using GCBI online software)

### GO functional enrichment analysis of all common DEGs

**Fig. 3:**
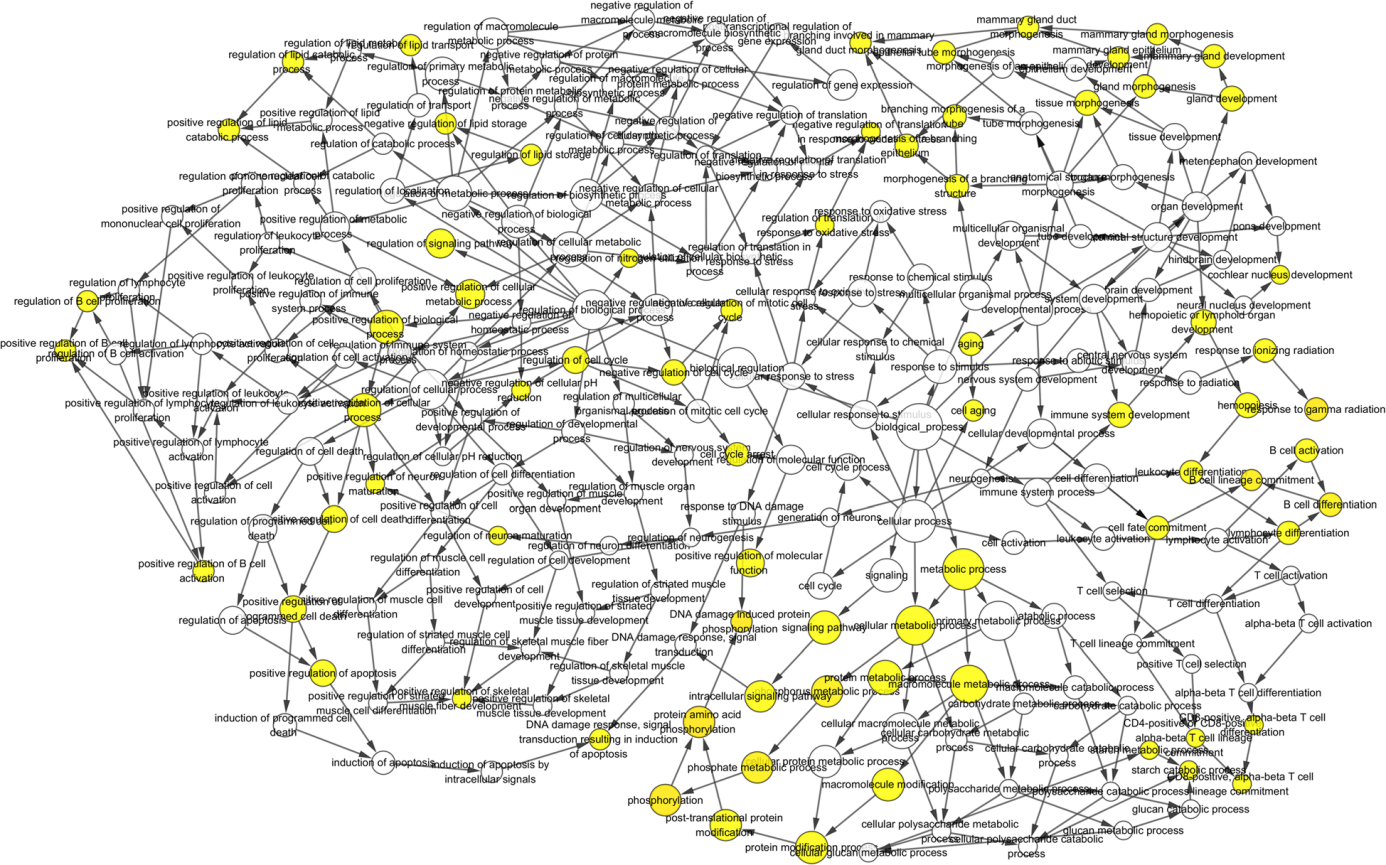
GO biological processes network of diferentially expressed genes in blood of Parkinson’s Disease from BiNGO software. Large nodes indicate more genes involved in. Yellow nodes: *P*-value < 0.05 and FDR < 0.05

**Fig. 4:**
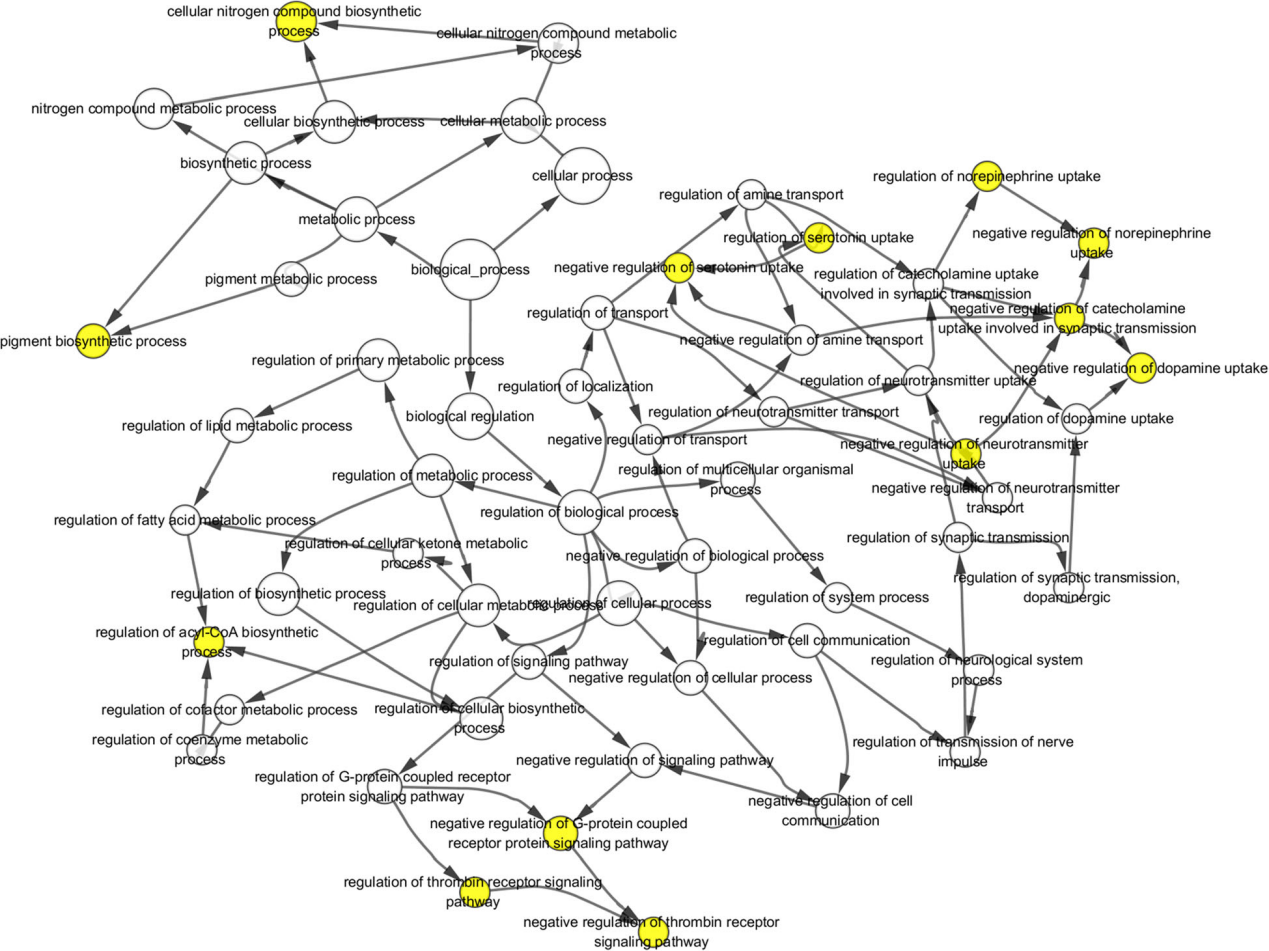
GO biological processes network of diferentially expressed genes in substantia nigra of Parkinson’s Disease from BiNGO software. Large nodes indicate more genes involved in. Yellow nodes: P-value < 0.05 and FDR < 0.05

The common DEGs from blood and brain SN were then subjected to enrichment analysis in BiNGO. The common DEGs in blood were enriched in biological processes (BP) of protein phosphorylation, DNA damage-induced protein phosphorylation, phosphorylation, response to gamma radiation and B cell lineage commitment, among other processes (Additional file 1: Table S1 and Fig. 3). DEGs from brain SN were enriched in BP including cellular nitrogen compound biosynthetic process, negative regulation of G-protein coupled receptor protein signaling pathway, pigment biosynthetic process, regulation of acyl-CoA biosynthetic process, negative regulation of neurotransmitter uptake and negative regulation of catecholamine uptake involved in synaptic transmission (Additional file 1: Table S2 and Fig. 4). In blood, these processes such as protein amino acid phosphorylation (9 genes), regulation of neuron maturation (1 gene), positive regulation of neuron maturation (1 gene) are highly related to neuron. SRPK2 and MAP2K6 are found to be involved in PD [33, 34]. BCL2 was reported in Alzheimer’s Disease [35]. In brain SN, negative regulation of neurotransmitter uptake (1 gene) and negative regulation of catecholamine uptake involved in synaptic transmission (1 gene) are highly related to neuron. SNCA is reported to be in PD [36]. All these information validate our finding regarding the association of these genes in PD.

### PPI network construction for common DEGs

**Fig. 5:**
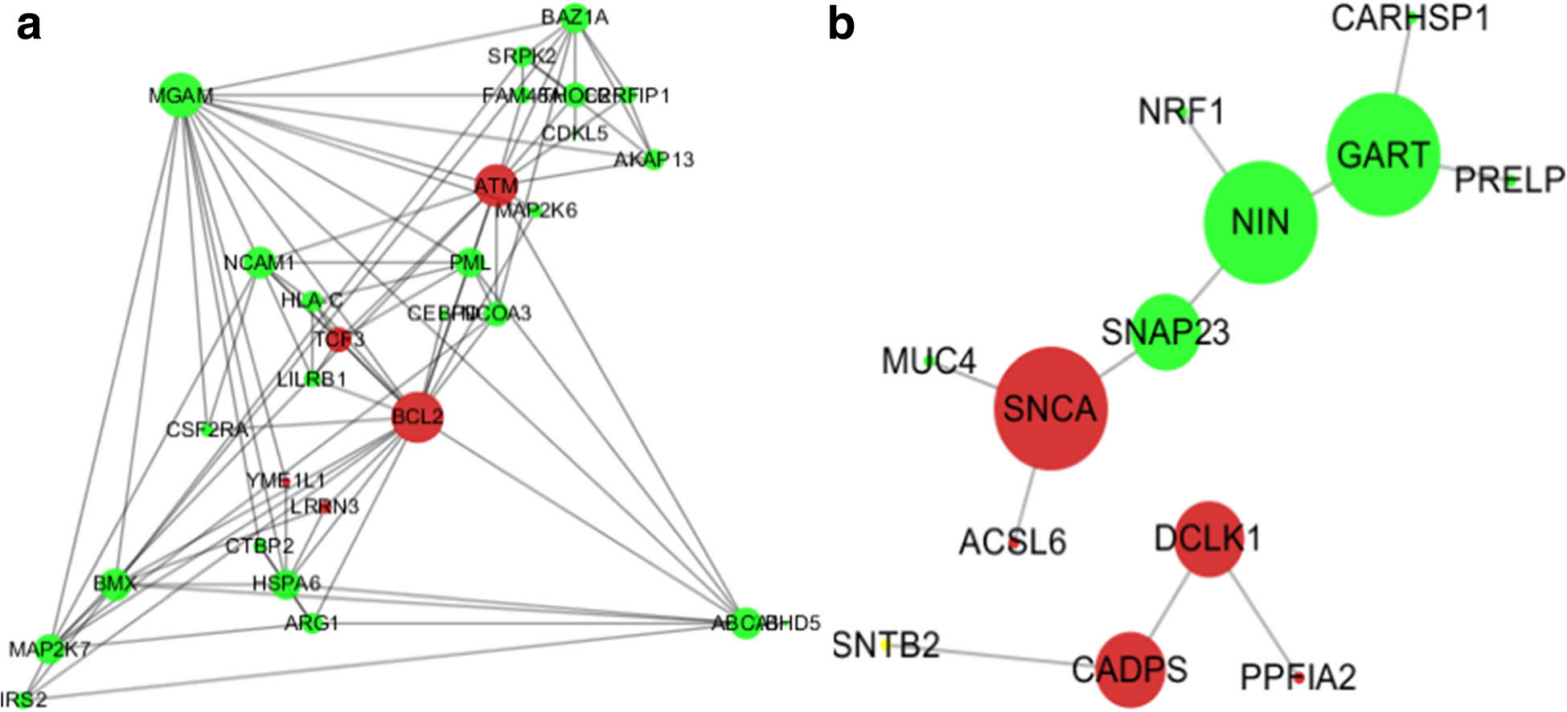
PPI networks obtained from Cytoscape software. **a** PPI network for common genes from blood; **b** PPI network for common genes from the brain substantia nigra region. Red nodes represent upregulated genes, and green nodes represent downregulated genes. Large nodes indicate bigger degrees

PPI networks were constructed using STRING v.10.5 database and then visualized and analyzed by Cytoscape software v.3.4.0. The PPI information obtained from STRING online software for common DEGs in blood is demonstrated in Additional file 1: Table S3 and the PPI network is shown in Fig. 5a. Here, the 29 genes exhibited a wide degree distribution with the highest degree of 18 and lowest degree of 1.The average degree value was found to be 6.14. Finally, the top three hub genes(i.e. top 10% of the total nodes) with higher degree were chosen, including ATM, MGAM and BCL2 genes. Out of the three hubs, one hub (BCL2) was already found to be related to Alzheimer’s Disease [35], which was further studied for their association in human PD. The PPI information obtained from STRING online software for common DEGs in brain SN is exhibited in Additional file 1: Table S4 and the PPI network is shown in Fig. 5b. We found that highest degree value was 3 and the lowest was 1 with an average of 1.69. In this analysis, the top three hub genes with higher degree included GART, SNCA and NIN genes. SNCA hub node was already found to be associated with human PD [36], and GART hub node has been verified in other neurodegenerative diseases such as Alzheimer’s Disease [37], but not in PD.

### Regulatory miRNAs associated with the common PD-specific genes and the analysis of lncRNAs associated with these miRNAs

To investigate the regulation of the PD-specific genes identified in our study by non-coding RNAs, miRNA associated with these genes were first retrieved from DIANA-Tarbase v.7.0 database, and then lncRNAs associated with these miRNAs were selected from DIANA-LncBase Experimentally v.2 (prediction score ≥ 0.90). The biological regulatory interaction of PD-specific genes identified in blood is exhibited in Table 4, which shows that four PD-specific genes were associated with several non-coding RNAs. Out of the miRNAs, 13 were previously known to be associated with several other neurodegenerative diseases, such as Alzheimer’s disease and nine were also previously associated with PD. The biological regulatory interaction of PD-specific genes identified in brain SN is showed in Table 5, which demonstrates that one PD-specific gene was associated with several non-coding RNAs. Out of the miRNAs, one was previously known to be associated with other neurodegenerative diseases and five were also previously associated with PD. Out of these five, four PD-specific genes (HSPA6, SRPK2, NOL7 and SNCA) were associated with miRNAs that were previously associated with PD. Moreover, most of these miRNAs associated with PD-specific genes were in turn regulated by lncRNAs. Among the miRNAs, 14 has been previously associated with neurodegenerative diseases, and 14 were previously associated with PD.

**Table 4:** Regulatory miRNAs associated with the common PD-specific genes identified in blood and the analysis of lncRNAs associated with these miRNAs

PD specific genes	miRNAs associated with genes	lncRNAs associated with the miRNAs
HSPA6	hsa-miR-204-3p^b^	LINC00999
	hsa-miR-548o-3p	CASC7, GABPB1-AS1, NEAT1, XIST
MAP2K6	hsa-miR-33a-5p^a^	KCNQ1OT1, MCF2L-AS1
	hsa-miR-590-3p^a^	CASC7, CTA-292E10.9, CTB-89H12.4, HCG11, LINC00657, LOC100507577, NEAT1, OIP5-AS1, OTUD6B-AS1, RP11-834C11.4, XIST
	hsa-miR-145-5p^a^	KCNQ1OT1,TUG1
	hsa-miR-425-5p	C1orf132, CTD-3025 N20.3, KCNQ1OT1, RP11-15H20.7, SNHG14, TTTY15, ZNRD1-AS1
	hsa-miR-1306-5p	KCNQ1OT1, SENP3-EIF4A1
	hsa-miR-148a-3p^a^	CASC7, KCNQ1OT1, OIP5-AS1, SNHG14
	hsa-miR-130a-3p^a^	CASC7, H19, SNHG14
	hsa-miR-148b-3p^a^	CASC7, OIP5-AS1, SLMO2-ATP5E, SNHG14
SRPK2	hsa-miR-3200-3p	BLOC1S5-TXNDC5, KCNQ1OT1, XIST
	hsa-miR-1292	
	hsa-miR-155-5p	CTD-2561 J22.5, RP11-175O19.4, XIST
	hsa-miR-15b-3p^a^	
	hsa-miR-17-3p^b^	RP3-323A16.1, XIST
	hsa-miR-181a-5p^b^	AC000403.4, CASC7, CTB-89H12.4, KCNQ1OT1, LINC00506, N4BP2L2-IT2, RP11-10E18.7, RP11-1134I14.8, RP11-147 L13.14, RP11-314B1.2, RP11-361F15.2, RP11-707A18.1, RP1-309I22.2, ZNF883, ZNRD1-AS1
	hsa-miR-181b-5p^b^	CASC7, CTB-89H12.4, KCNQ1OT1, RP11-1134I14.8, XIST
	hsa-miR-181c-5p^b^	CTB-89H12.4, KCNIP4-IT1, KCNQ1OT1, RP11-1134I14.8
	hsa-miR-181d^b^	
	hsa-miR-183-3p^a^	
	hsa-miR-1976	HNRNPUL2-BSCL2, KCNQ1OT1, LOC100129917, NEAT1, TSIX
	hsa-miR-19a-3p^b^	FAM201A, H19, KCNA3, KCNQ1OT1, LINC00094, RP11-337C18.8, RP11-523G9.3, SNHG14
	hsa-miR-19b-3p^b^	CASC7, FAM201A, H19, KCNA3, KCNQ1OT1, LINC00094, RP11-337C18.8, RP11-523G9.3, SNHG14
	hsa-miR-21-5p^a^	
	hsa-miR-320a	ALMS1-IT1, CASC7, CTB-36H16.2, CTB-89H12.4, KCNIP4-IT1, LINC00663, LMCD1-AS1, MALAT1, NEAT1, RP11-145P16.3, XIST
	hsa-miR-3685	KCNQ1OT1
	hsa-miR-3689a-3p	TTTY15
	hsa-miR-4518	
	hsa-miR-944	
NOL7	hsa-miR-199b-3p^a^	CTB-89H12.4, ERVK3–1, XIST
	hsa-miR-199a-3p^b^	CTB-89H12.4, ERVK3–1, XIST
	hsa-miR-328	
	hsa-miR-129-5p^a^	CASC7, ERVK3–1, KCNQ1OT1, MALAT1, NEAT1,
	hsa-miR-374a-5p^a^	CTC-444 N24.11, CTD-2561 J22.5, RP11-613D13.5, TRG-AS1, XIST, ZNRD1-AS1
	hsa-miR-744-5p	FLJ16779,
	hsa-miR-374b-5p^a^	CTA-292E10.9, CTC-444 N24.11, OIP5-AS1, RP11-221 J22.1, RP11-38P22.2, XIST,
	hsa-miR-548o-3p	CASC7, GABPB1-AS1, NEAT1, XIST,

^a^Previously associated with several neurodegenerative diseases, but not PD

^b^Previously associated with PD

**Table 5:** Regulatory miRNAs associated with the common PD-specific genes identified in substantia nigra and the analysis of lncRNAs associated with these miRNAs

Genes associated with PD	miRNAs associated with genes	lncRNAs associated with the miRNAs
SNCA	hsa-miR-93-3p^b^	AC012065.7,KCNQ1OT1, LINC00342
	hsa-miR-153^b^	
	hsa-miR-23b-3p^b^	CASC7,CTC-459F4.3, KCNQ1OT1, RP11-215G15.5, SNHG14, TOB1-AS1, XIST, ZNRD1-AS1
	hsa-miR-500a-5p	SNHG22
	hsa-miR-34a-5p^b^	AC004951.6, AC092535.3, KCNQ1OT1, LINC00662, PCBP2-OT1, RP11-693 J15.5
	hsa-miR-29a-3p^a^	AC005154.6, H19, KCNQ1OT1, LINC00674, MIR4697HG, NEAT1, RP11-314B1.2, RP11-582E3.6, RP4-630A11.3, THUMPD3-AS1, TTTY15, TUG1
	hsa-miR-7-5p^b^	AC005154.6, DLX6-AS1, KCNQ1OT1, LINC01233, LINC01314, RP11-679B19.1, XIST
	hsa-miR-181a-2-3p	KCNQ1OT1, NEAT1

^a^Previously associated with several neurodegenerative diseases, but not PD

^b^Previously associated with PD

### mTF-miRNA-gene-gTF regulatory network construction of PD-specific genes

**Fig. 6:**
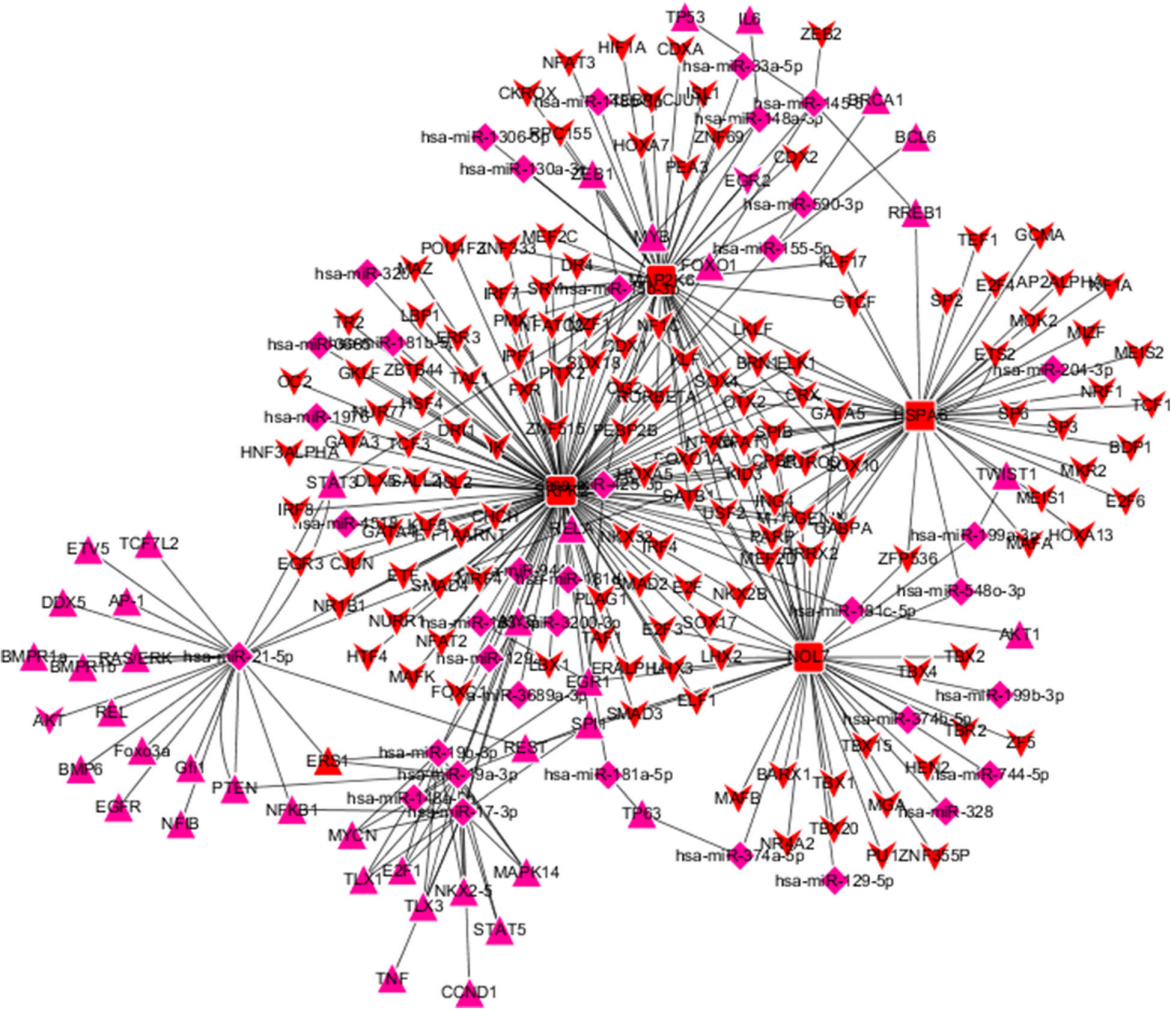
The mTF-miRNA-gene-gTF regulatory network of PD-specific genes identified in blood datasets obtained from Cytoscape software. The diamond-shaped magenta nodes represent miRNAs, the triangle-shaped magenta nodes represent transcription factors (TFs) associated with these miRNAs (mTFs), and the round rectangle-shaped red nodes represent the PD-specific genes. The V-shaped red nodes represent the TFs associated with these genes (gTFs)

**Fig. 7:**
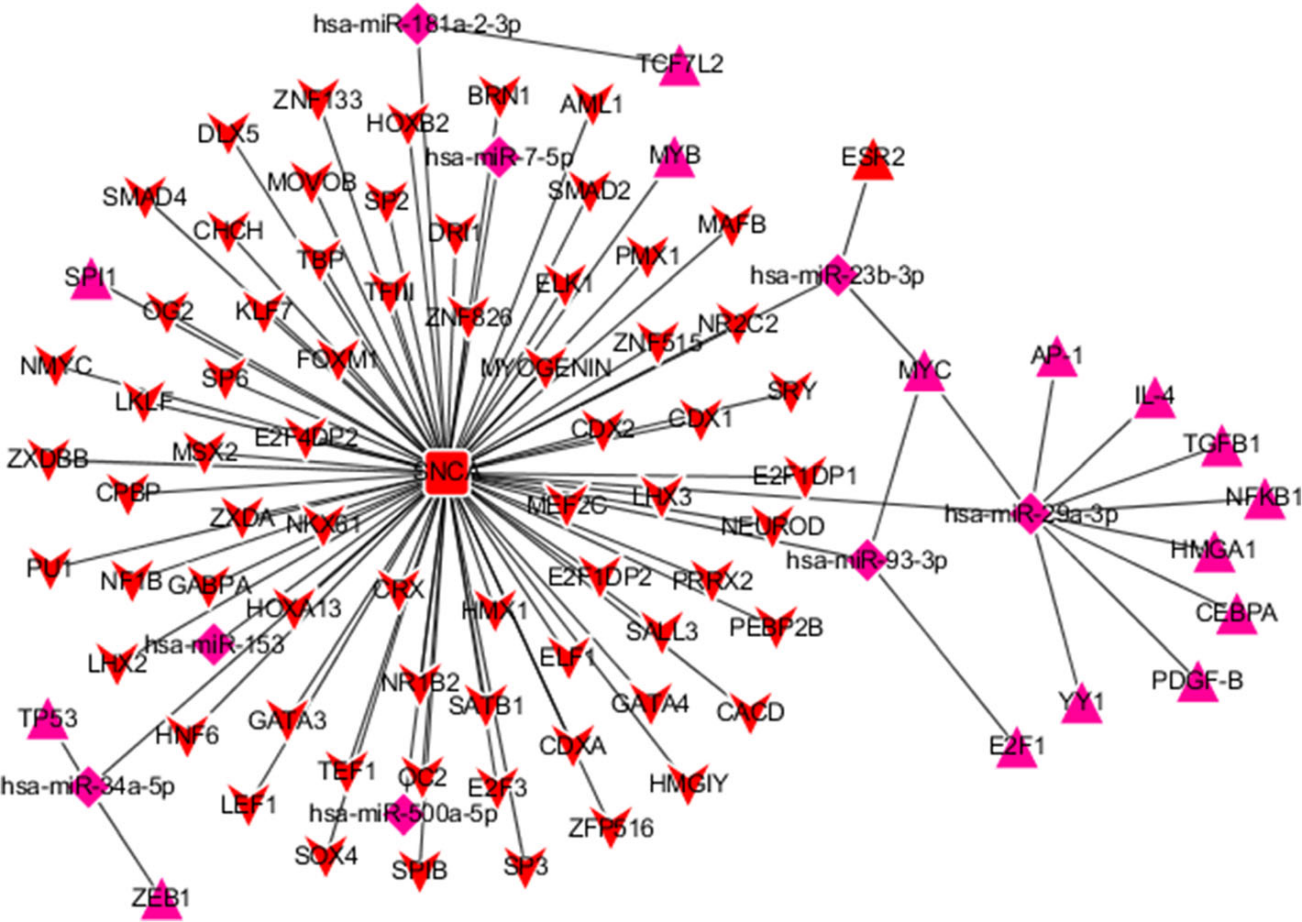
The mTF-miRNA-gene-gTF regulatory network of PD-specific genes identified in substantia nigra datasets obtained from Cytoscape software. The diamond-shaped magenta nodes represent miRNAs, the triangle-shaped magenta nodes represent transcription factors (TFs) associated with these miRNAs (mTFs), and the round rectangle-shaped red nodes represent the PD-specific genes. The V-shaped red nodes represent the TFs associated with these genes (gTFs)

To further study the regulatory mechanism of these common PD-specific genes (the genes from Tables 2 and 3 that were defined as PD-specific by authors) in PD pathogenesis, we constructed a regulatory network comprising common PD-specific genes, the TFs associated with these genes (gTFs), miRNAs associated with these genes and the TFs associated with these miRNAs (mTFs). Additional file 1: Table S5 and Fig. 6 show the mTF-miRNA-gene-gTF regulatory networks identified in blood. Additional file 1: Table S6 and Fig. 7 show the mTF-miRNA-gene-gTF regulatory networks identified in brain SN.

### IncRNAs and mTFs associated with these PD-specific miRNAs related to the PD-specific genes

The 14 PD-specific miRNAs shown in Tables 4 and 5 (hsa-miR-204-3p, hsa-miR-17-3p, hsa-miR-181a-5p, hsa-miR-181b-5p, hsa-miR-181c-5p, hsa-miR-181d, hsa-miR-19a-3p, hsa-miR-19b-3p, hsa-miR-93-3p, hsa-miR-153, hsa-miR-23b-3p, hsa-miR-34a-5p, hsa-miR-9-5p and hsa-miR-7-5p) were further analyzed in the DIANA-LncBase Experimental v.2 database to examine the regulatory information of miRNAs-lncRNAs (Table 6) and in the TransmiR v.1.2 database to examine the regulatory information of miRNAs-mTFs. The 14 miRNAs were found to be associated with 45 lncRNAs. These 14 miRNAs control four PD-specific genes in our study, and these miRNAs are in turn regulated by 31mTFs (Table 6). By combining all the regulatory information obtained from our results, we constructed a regulatory pattern involving the PD-specific miRNAs, lncRNAs, PD-specific genes and mTFs.

**Table 6:** The lncRNA-mediated PD-specific miRNAs associated the PD-specific mRNAs identified in our study regulatory network

Tissue	PD-specific miRNA	lncRNAs associated the miRNAs	the PD-specific mRNAs associated the miRNA	TFs associated with the miRNAs
Blood	hsa-miR-204-3p	LINC00999	HSPA6	
	hsa-miR-17-3p	RP3-323A16.1, XIST	SRPK2	Activation:CCND1,E2F1,MYC, MYCN, NKX2–5, TLX1, TLX3, TNF, ERS1, STAT5; Repression: NFKB1, SPI1; Neutral: MAPK14
	hsa-miR-181a-5p	AC000403.4, CASC7, CTB-89H12.4, KCNQ1OT1, LINC00506, N4BP2L2-IT2, RP11-10E18.7, RP11-1134I14.8, RP11-147 L13.14, RP11-314B1.2, RP11-361F15.2, RP11-707A18.1, RP1-309I22.2, ZNF883, ZNRD1-AS1	SRPK2	Neutral: TP63
	hsa-miR-181b-5p	CASC7, CTB-89H12.4, KCNQ1OT1, RP11-1134I14.8, XIST	SRPK2	
	hsa-miR-181c-5p	CTB-89H12.4, KCNIP4-IT1, KCNQ1OT1, RP11-1134I14.8	SRPK2	Activation: AKT1
	hsa-miR-181d		SRPK2	
	hsa-miR-19a-3p	FAM201A, H19, KCNA3, KCNQ1OT1, LINC00094, RP11-337C18.8, RP11-523G9.3, SNHG14	SRPK2	Activation: E2F1, MYC, MYCN, NKX2–5, TLX1, TLX3, ERS1, STAT5; Repression:SPI1; Neutral:PTEN, MAPK14
	hsa-miR-19b-3p	CASC7, FAM201A, H19, KCNA3, KCNQ1OT1, LINC00094, RP11-337C18.8, RP11-523G9.3, SNHG14	SRPK2	Activation: E2F1, MYC, MYCN, NKX2–5, TLX1, TLX3, ERS1; Neutral: MAPK14
	hsa-miR-199a-3p	CTB-89H12.4, ERVK3–1, XIST	NOL7	Neutral: TWIST1
Substantia nigra	hsa-miR-93-3p	AC012065.7,KCNQ1OT1, LINC00342	SNCA	Repression: MYC; Neutral: E2F1
	hsa-miR-153		SNCA	
	hsa-miR-23b-3p	CASC7,CTC-459F4.3, KCNQ1OT1, RP11-215G15.5, SNHG14, TOB1-AS1, XIST, ZNRD1-AS1	SNCA	Repression: MYC; Neutral: ESR2
	hsa-miR-34a-5p	AC005154.6, H19, KCNQ1OT1, LINC00674, MIR4697HG, NEAT1, RP11-314B1.2, RP11-582E3.6, RP4-630A11.3, THUMPD3-AS1, TTTY15, TUG1	SNCA	Activation: HMGA1, AP-1; Repression: MYC, NFKB1, YY1, IL-4, PDGF-B, TGFB1; Neutral: CEBPA
	hsa-miR-7-5p	CTB-89H12.4, KCNQ1OT1, RP11-273G15.2, RP11-314B1.2, RP11-793H13.8, SNHG14, TSNAX-DISC1, TUG1	SNCA	Activation: NFKB1, IL1B, TLR2, TLR4, TLR7, TLR8, TNF, MYC, NFKB1; Repression: TLX

### mTF-miRNA-gene-gTF regulatory analysis of the common non-PD-specific genes

To study the regulatory interaction of the common non-PD-specific genes, we got mTF-miRNA-gene-gTF regulatory patterns from blood (Additional file 1: Table S7) and brain SN (Additional file 1: Table S8) respectively. We obtained 359 gTFs (score = 1) that were related to 26 common DEGs from blood datasets. The TCF3, BAZ1A and THOC2 genes were regulated by the highest numbers of gTFs (105, 95 and 95 gTFs, respectively). Previous reports showed TCF3 involvement in PD [38]. TCF3, a transcriptional repressor [39], is counteracted at multiple levels by Wnt signaling [40]. Alteration in normal Wnt signaling pathway has been already found in gene expression studies of blood of PD patients [40]. It was found that BAZ1A depletion influence the expression of genes important for nervous system development and function [41]. THOC2, an abundant protein in the developing and mature human [42, 43] and adult mouse brains [44]. THOC2 depletion has been already reported to stimulate neurite outgrowth in primary rat hippocampal neurons [44]. It has been identified also to be involved in neoplasms and multiple sclerosis [45, 46].

The common non-PD-specific genes were associated with 100 experimentally validated miRNAs. Out of these miRNAs, 25 were previously known to be associated with PD (Table 7). The NCOA3, AKAP13 and BCL2 genes were regulated by the highest numbers of miRNAs (21, 19 and 14 miRNAs, respectively). NCOA3, a nuclear receptor coactivator, has been reported to play an important role in some biological processes, such as cell proliferation and apoptosis [47]. Previous study indicate that high expression of NCOA3 can suppress cells apoptosis induced by histone deacetylase inhibitor in breast cancer [47]. BCL2, an anti-apoptotic gene, regulate cell death (apoptosis), by either inducing or inhibiting apoptosis. The observed upregulation of BCL2 by GDNF suggests that BCL2 protects neurons, possibly by regulating cell apoptosis. Previous studys have shown its alteration in Alzheimer’s Disease [35]. AKAP13 protein functions as an anchor protein for the regulatory subunit of protein kinase A which is reported to increase tau phosphorylation. AKAP13 are likely more specifically involved in tau phosphorylation pathways that is highly related to Alzheimer’s disease [48, 49]. Out of these three genes, NCOA3 was associated with hsa-let-7 g-5p, hsa-miR-106b-5p, hsa-miR-17-5p, hsa-miR-181c-5p, hsa-miR-20a-5p, hsa-miR-25-3p and hsa-miR-27b-3p that were reported to be previously known to be associated with PD (Table 7). AKAP13 was associated with hsa-miR-34a-5p, and hsa-miR-34c-5p that were reported to be previously known to be associated with PD (Table 7). BCL2 was associated with hsa-miR-132–5p, hsa-miR-182–5p, hsa-miR-23b-3p and hsa-miR-34a-5p that were reported to be previously known to be associated with PD (Table 7). Overall, the 100 miRNAs were identified to be associated with 98 mTFs.

**Table 7:** miRNAs that are previously known to be associated with PD associated with non-PD-specific genes identified in our study

miRNAs from blood	mRNA targets associated with the miRNAs	miRNAs from substantia nigra	mRNA targets associated with the miRNAs
hsa-let-7 g-5p	NCOA3	hsa-let-7a-5p	SNAP23
hsa-let-7i-5p	IRS2	hsa-let-7e-5p	SNAP23
hsa-miR-101-5p	TMX4	hsa-miR-106a-5p	NIN
hsa-miR-106a-5p	NCOA3	hsa-miR-106b-5p	NIN
hsa-miR-106b-5p	ABHD5, NCOA3	hsa-miR-125a-5p	NIN
hsa-miR-125a-5p	MAP2K7	hsa-miR-17-5p	SNTB2
hsa-miR-132-3p	ATM	hsa-miR-182–5p	SNTB2
hsa-miR-132–5p	BCL2	hsa-miR-20a-5p	NIN
hsa-miR-143-3p	FAM102A	hsa-miR-23b-3p	NIN, SNTB2
hsa-miR-17-5p	ABHD5, NCOA3	hsa-miR-25-3p	SNTB2
hsa-miR-181c-5p	NCOA3	hsa-miR-302d-5p	SNTB2
hsa-miR-182–5p	BCL2	hsa-miR-30a-5p	SNTB2
hsa-miR-18a-5p	ATM	hsa-miR-30e-5p	SNTB2
hsa-miR-20a-5p	ABHD5, NCOA3	hsa-miR-93-5p	SNTB2
hsa-miR-212-3p	LRRFIP1	hsa-miR-9-5p	SNTB2
hsa-miR-214-3p	FAM120A		
hsa-miR-23b-3p	BCL2		
hsa-miR-25-3p	NCOA3		
hsa-miR-27a-3p	ABCA1, FAM102A, FAM120A		
hsa-miR-27b-3p	NCOA3		
hsa-miR-30e-5p	IRS2		
hsa-miR-34a-5p	AKAP13, ATM, BCL2		
hsa-miR-34c-5p	AKAP13		
hsa-miR-363-3p	IRS2		
hsa-miR-93-5p	ABCA1, ABHD5		

In brain SN, 333 gTFs were associated with 14 common genes. The NIN, PPFIA2 and ATP5S genes were regulated by the highest numbers of gTFs (139, 102 and 93 gTFs, respectively). NIN, a large coiled-coil protein, is essential for neurogenesis, angiogenesis and stem cell fate [50]. Previous study showed that alternative splicing in NIN gene appears sufficient for neural stem cells differentiation into neurons [50]. PPFIA2 is a member of the leukocyte common antigen-related PTP interacting protein family liprin [47]. It is known to be downregulated by androgens in prostate cancer cell lines [47] and is found to play crucial functions in the pathophysiology of schizophrenia and bipolar disorder [51]. ATP5S gene encodes a subunit of mitochondrial ATP synthase which utilizes the electro chemical gradient to synthetize ATP from ADP in inner mitochondrial membrane by oxidative phosphorylation [52]. Mitochondrial dysfunction is reported to be a pathological pathway associated with PD [53]. But little is known about the involvement of ATP5S in PD.

The common non-PD-specific genes were associated with 50 experimentally validated miRNAs. Out of these miRNAs, 16 were previously known to be associated with PD (Table 7). It was found that NIN and SNTB2 were regulated by the highest numbers of miRNAs (18 and 25 miRNAs, respectively), and that NIN and SNTB2 were associated with 16 miRNAs (such as miR-9-5p) (Table 7) that were previously associated with PD. For example, in study, miR-9-5p increased by more than three times in treated PD patients compared with those of controls [54]. Moreover, miR-9-5p were in turn regulated by mTFs of interleukin 1 beta (IL1B) and nuclear factor kappa B subunit 1 (NFKB1). Genetic variation in the proinflammatory cytokine gene IL1B can contribute to risk of developing PD. These finding supported that miR-9-5p may contributes to the pathogenesis of sporadic PD. Previous finding reported SNTB2 involved in atherosclerosis [55], and that its risk factors in atherosclerosis include higher plasma urate level [56] which has linked to lower risk of PD in men [57]. Overall, the 50 miRNAs were identified to be associated with 63 mTFs.

These data support the finding that these genes may be significant factors in PD, but furture studys are needed.

### SNP analysis of the common PD-specific and other neurodegenerative disease-specific genes

We next obtained SNPs corresponding to the common DEGs (both the PD-specific and the neurodegenerative disease-specific genes) of our study from the online databases MirSNP, miRdSNP and LincSNP 2.0. Six miRNAs were found to be associated with two PD-specific SNPs corresponding to the PD-specific gene SNCA (Table 8). Two SNPs were associated with these six miRNAs (Table 8). The chromosome locus and allele gene for these two SNPs were then searched using the dbSNP database. These two SNPs were located on chromosome 4.

**Table 8:** SNPs in PD with associated PD-specific genes and miRNAs

MicroRNAs	SNPs	Allele	Chromosome	Gene	MinorAlleleCount	Experimentally_confirmed
hsa-miR-141	rs17016074	A/G	4:89726127	SNCA	A = 0.0503/252	
hsa-miR-153	rs17016074	A/G		SNCA		Yes
hsa-miR-223	rs17016074	A/G		SNCA		
hsa-miR-499-3p	rs17016074	A/G		SNCA		
hsa-miR-504	rs17016074	A/G		SNCA		
hsa-miR-7	rs17016074	A/G		SNCA		Yes
hsa-miR-141	rs356165	A/G	4:89725735	SNCA	A = 0.4842/2425	
hsa-miR-153	rs356165	A/G		SNCA		Yes
hsa-miR-223	rs356165	A/G		SNCA		
hsa-miR-504	rs356165	A/G		SNCA		
hsa-miR-7	rs356165	A/G		SNCA		Yes

Twenty-three miRNAs were found to be associated with seven neurodegenerative disease-specific SNPs corresponding to four neurodegenerative disease-specific genes (Table 9). Seven SNPs were associated with these 23 miRNAs. The chromosome locus and allele gene for these seven SNPs were then searched using the dbSNP database. The chromosome loci of these SNPs include 6, 14, 16 and 18.

**Table 9:** SNPs with their associated miRNAs and genes in neurodegenerative diseases other than PD

MicroRNAs	SNPs	Allele	Chromosome	Gene	MinorAlleleCount	Region	SNPs related diseases by experimentally_confirmed
hsa-miR-4428	rs1049853	C/T	6:31269123	HLA-C	A = 0.0903/452	3’UTR	Alzheimer’s disease, Rheumatoid arthritis
hsa-miR-27a-5p	rs1016860	A/G	18:63127841	BCL2	*T* = 0.1166/584	3’UTR	Multiple sclerosis
hsa-miR-3127-5p	rs1016860	A/G	18:63127841	BCL2	T = 0.1166/584	3’UTR	
hsa-miR-3158-3p	rs1016860	A/G	18:63127841	BCL2	T = 0.1166/584	3’UTR	
hsa-miR-4720-3p	rs1016860	A/G	18:63127841	BCL2	T = 0.1166/584	3’UTR	
hsa-miR-4789-5p	rs1016860	A/G	18:63127841	BCL2	T = 0.1166/584	3’UTR	
hsa-miR-629-5p	rs1016860	A/G	18:63127841	BCL2	T = 0.1166/584	3’UTR	
hsa-miR-1224-5p	rs1058929	A/G/T	16:8853394	CARHSP1	C = 0.4740/2374	3’UTR	Alzheimer’s disease
hsa-miR-1265	rs1058929	A/G/T	16:8853394	CARHSP1	C = 0.4740/2374	3’UTR	
hsa-miR-3605-5p	rs1058929	A/G/T	16:8853394	CARHSP1	C = 0.4740/2374	3’UTR	
hsa-miR-3915	rs1058929	A/G/T	16:8853394	CARHSP1	C = 0.4740/2374	3’UTR	
hsa-miR-4710	rs1058929	A/G/T	16:8853394	CARHSP1	C = 0.4740/2374	3’UTR	
hsa-miR-5585-5p	rs1058929	A/G/T	16:8853394	CARHSP1	C = 0.4740/2374	3’UTR	
hsa-miR-193a-5p	rs9953	G/T	16:8853271	CARHSP1	G = 0.4507/2257	3’UTR	Alzheimer’s disease, glioblastoma
hsa-miR-3190-3p	rs9953	G/T	16:8853271	CARHSP1	G = 0.4507/2257	3’UTR	
hsa-miR-3615	rs9953	G/T	16:8853271	CARHSP1	G = 0.4507/2257	3’UTR	
hsa-miR-335-5p	rs1058967	A/G	16:8853179	CARHSP1	C = 0.4744/2376	3’UTR	Alzheimer’s disease
hsa-miR-451b	rs1058967	A/G	16:8853179	CARHSP1	C = 0.4744/2376	3’UTR	
hsa-miR-4533	rs1058967	A/G	16:8853179	CARHSP1	C = 0.4744/2376	3’UTR	
hsa-miR-4797-5p	rs1058967	A/G	16:8853179	CARHSP1	C = 0.4744/2376	3’UTR	
hsa-miR-3679-3p	rs2447924	G/T	16:8854616	CARHSP1	C = 0.4754/2381	3’UTR	Alzheimer’s disease
hsa-miR-4286	rs2447924	G/T	16:8854616	CARHSP1	C = 0.4754/2381	3’UTR	
hsa-miR-4517	rs8017316	C/G	14:50322860	ATP5S	G = 0.4511/2259	Intron	Alzheimer’s disease

## Discussion

### Meta-analysis of common genes

In the past decades, microarray has been widely used to identify DEGs and pathways underlying PD pathogenesis. In these studies, most microarray datas are mainly from the brain regions or blood in PD. The analysis of this brain regions from PD may only highlight genes associated with changes in cellular composition [58], However, brain tissues are not easily obtained. In recent years, there is evidence to prove that low concentrations of urate in the blood serum increased the risk of PD [12], and that there is a growing interest in the discovery of blood biomarkers for PD. Some genes have been proved to take part in important neurodegeneration molecular pathways in PD patients.

A large number of microarray gene expression studies on PD have been performed, but some of the results showed low consistency among involved genes and pathways. Meta-analysis has suggested new biological insights, as well as identification of greater consistent genes and pathways potential in PD pathogenesis [13, 15, 16]. Numbers of meta-analysis on PD have been conducted, with few focused on understanding the regulatory network involving genes, miRNAs, transcription factors (TFs) and lncRNA in PD progression. Moreover, few meta-analysis on PD is focused on comparative analysis of microarray datases profiles from brain and blood samples.

In our study, meta-analysis merges several datasets from PD brain SN or blood into a single analysis to obtain more meaningful set of DEGs and these DEGs are analyzed in various biogenetic databases to get related non-coding RNAs and gTFs, among others. In this study of DEGs in PD patients compared with healthy controls, we identified 36 common DEGs in blood studies and 17 common DEGs in five brain SN studies using meta-analysis technique-Vote counting generic ways. Of the total common genes, 28 genes were previously reported as associated with other neurodegenerative diseases (see Tables 2 and 3), but not previously known to be associated with PD, and five genes were previously shown to be associated with PD. Among the 28 genes associated with other neurodegenerative diseases, 22 were downregulated and six were upregulated in PD compared with controls. We further studied these 28 genes for their association with PD. Out of the five genes previously associated with PD, three genes (HSPA6, MAP2K6 and SRPK2 genes) were downregulated and two genes (NOL7 and SNCA genes) were upregulated in PD compared with controls. A recent study reported the role of HSPA6 in SH-SY5Y neuronal cells which are used as a model system for neurodegenerative diseases such as Alzheimer’s disease and PD [59]. HSPA6, aslo known as Hsp70B’, is a member of heat shock proteins (Hsps) 70 family and the Hsp70 family of heat shock proteins has been well known for its roles in cytoprotective effects against cell death and implicated in neuroprotection [59]. It was also found that HSPA6 has previously been examined in human neurodegenerative diseases and was proposed as a potential treatment strategy to counter PD [60]. Studies have found that MAP2K6 binds to and regulates the expression of the PD-related protein LRRK2, which is a prevalent cause of sporadic PD [33]. SRPK2, a serine/arginine protein-specific kinase, is highly expressed in the brain. Increased amounts of SRPK2 can lead to the hyperphosphorylation of serine-arginine-rich proteins, which in turn induces changes in alternative pre-mRNA splicing observed in PD [34]. NOL7 is a candidate cancer suppressor that localizes to 6p23, a segment with frequent loss of heterozygosity (LOH) in many tumors [61]. NOL7 interacts with amyloid precursor protein (APP) protein which accumulates in mitochondrial membrane in PD, and that APP interacts with LRRK2 and then is phosphorylated at Thr668 within its intracellular domain to promote neurotoxicity in PD [62]. SNCA was reported to be associated with PD, and different SNPs in the SNCA gene had a correlation with increased or decreased risk of PD [63]. All these data help validate our results showing an association of these genes in PD.

Moreover, our study screen the overlapped genes among common DEGs obtained from two different tissues (brain SN and blood), but did not get any common genes. And It was found that the common DEGs from blood and brain SN are enriched in different BP.

### The analysis of lncRNAs and mTFs associated with these PD-specific miRNAs related to the PD-specific genes

The biological regulations of the common PD-specific genes are shown in Tables 4 and 5. We examined the relevance of experimentally validated miRNAs and lncRNAs with these five PD genes. Our analysis showed that four out of the five PD-specific genes (HSPA6, SRPK2, NOL7 and SNCA) are regulated by 14 PD-specific miRNAs (Tables 4 and 5). Interestingly, SRPK2 (obtained from blood) was regulated by the highest number of PD-specific miRNAs (hsa-miR-17-3p, hsa-miR-181a-5p, hsa-miR-181b-5p, hsa-miR-181c-5p, hsa-miR-181d, hsa-miR-19a-3p and hsa-miR-19b-3p) and these seven PD-specific miRNAs are regulated by 25 lncRNAs and 16 mTFs (Table 6).

### mTF-miRNA-gene-gTF regulatory network analysis

In this study, we screened the overlapped gTFs of PD-specific and non-PD-specific genes mTF-miRNA-gene-gTF regulatory network obtained from blood studies. Result showed that 135 gTFs are found to be involved in these two networks. It was found that core promoter element-binding protein (CPBP), Spi-B transcription factor (SPIB) and KID3 are the gTFs, which regulated a maximum number of genes, 28, 27 and 24 genes respectively. The CPBP was associated with four PD-specific genes (HSPA6, MAP2K6, SRPK2 and NOL7) and 24 non-PD-specific genes as obtained from TRANSFAC database [27]. SPIB was found to be associated with four PD-specific genes(HSPA6, MAP2K6, SRPK2 and NOL7) and 23 non-PD-specific genes. KID3 was found to be associated with four PD-specific genes(HSPA6, MAP2K6, SRPK2 and NOL7) and 20 non-PD-specific genes in GeneCards database (http://www.genecards.org/). CPBP is reported to be with lung cancer [64] and SPIB is found to be associated with leukemia cells [65]. Previous study reported that KID3 was to be highly expressed in adult brain [66]. These three gTFs are not reported previously to be associated with PD. But they regulate the four PD-specific genes and many non-PD-specific genes from blood.

In PD-specific and non-PD-specific genes mTF-miRNA-gene-gTF regulatory network obtained from brain SN studies, 59 overlapped gTFs is found to be involved in these two networks. CPBP, caudal type homeobox 1(CDX1) and SPIB are the gTFs, which regulated maximum genes i.e. 13, 12 and 12 genes, respectively. All three gTFs were found to regulate the PD-specific gene SNCA and 12, 11, and 11 non-PD-specific genes respectively.

The overlapped gTFs were filtered out in PD-specific regulatory patterns from blood and brain SN and 42 gTFs were screened. CPBP and SPIB are the gTFs, which regulated a maximum number of genes, 5 PD-specific genes(HSPA6, MAP2K6, SRPK2, NOL7 and SNCA) respectively.

Finally, we screened the overlapped gTFs in non-PD-specific regulatory patterns from blood and brain SN, 236 gTFs were identified. CPBP, KID3 and SPIB are the gTFs, which regulated maximum genes i.e. 36, 34 and 34 genes.

Combination of above results, we supposed that CPBP and SPIB may play significant roles in PD. But there is no verification of experiments. So further researches are needed.

Next, analysis of these miRNAs associated with PD-specific genes and non-PD-specific genes resulted in the identification of common PD-specific miRNAs (hsa-miR-181c-5p, hsa-miR-23b-3p and hsa-miR-34a-5p). Hsa-miR-181c-5p was shown to regulate PD-specific gene SRPK2 from blood (shown in Table 4) and regulate non-PD-specific gene NCOA3 from blood (Table 7). Hsa-miR-23b-3p is found to regulate PD-specific gene SNCA from brain SN (shown in Table 4), and regulate non-PD-specific gene BCL2 from blood and NIN and SNTB2 from brain SN (Table 7). It was found that hsa-miR-34a-5p regulate PD-specific gene SNCA from brain SN (shown in Table 4), and regulate non-PD-specific gene AKAP13, ATM and BCL2 from blood and SNTB2 from brain SN (Table 7). Maybe, these three common miRNAs play an important role in PD, but further study is needed.

The biological regulations of the common PD-specific genes are shown in Tables 4 and 5. We examined the relevance of experimentally validated miRNAs and lncRNAs with these five PD genes. Our analysis showed that four out of the five PD-specific genes (HSPA6, SRPK2, NOL7 and SNCA) are regulated by 14 PD-specific miRNAs (Tables 4 and 5). Interestingly, SRPK2 was regulated by the highest number of PD-specific miRNAs (hsa-miR-17-3p, hsa-miR-181a-5p, hsa-miR-181b-5p, hsa-miR-181c-5p, hsa-miR-181d, hsa-miR-19a-3p and hsa-miR-19b-3p) and these seven PD-specific miRNAs are regulated by 25 lncRNAs and 16 mTFs (Table 6).

### Identification of feed forward loops from mTF-miRNA-gene-gTF regulatory networks

Analysis of the regulatory network of PD-specific genes identified in blood datasets revealed the presence of two interesting feed-forward loops (FFLs), in which a TF regulates a miRNA and in turn they both regulate a target gene. One FFL was detected among the SPRK2 gene, hsa-miR-19a-3p and Spi-1 proto-oncogene (SPI1). The TransmiR information indicated that hsa-miR-19a-3p is inhibited by the SPI1 TF. By combing the TransmiR and DIANA-Tarbase data, we found that SPI1 and hsa-miR-19a-3p both regulate the expression of its target gene SPRK2. Studies have found that SPI1 plays an important role in the regulation of genes related to the function of microglia, which accumulate in PD [67]. Another study revealed that lower SPI1 expression reduces risk for AD by regulating myeloid gene expression and cell function [68]. Another study revealed a function of miR-19a in multiple system atrophy, which is a sporadic neurodegenerative disease, identifying lower expression of this miRNA in multiple system atrophy patients than controls [69]. Moreover, previous study reported the association of downregulated levels of miR-19a in idiopathic Parkinson’s disease (IPD) [70]. This information provides functional insights into the finding of downregulation of hsa-miR-19a-3p by SPI1. However, the FFL gene SPRK2 was identified downregulated in our study.

**Fig. 8:**
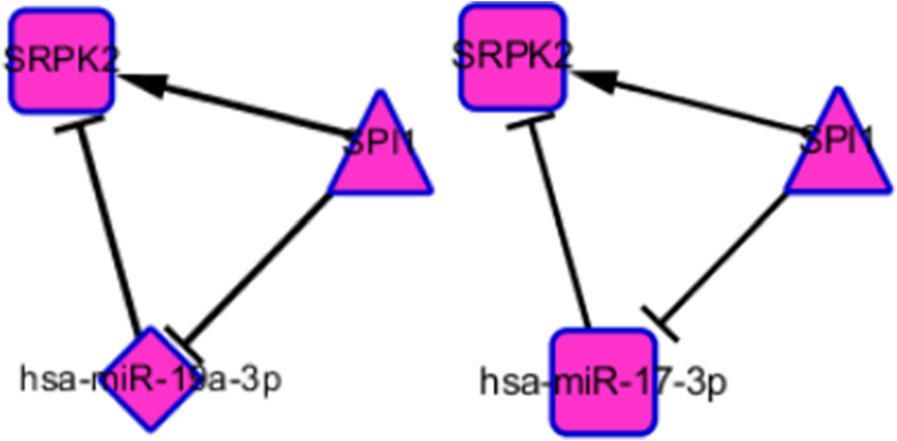
The feed forward loop from mTF-miRNAgene-gTF regulatory networks obtained from Cytoscape software. In this network, the diamond shaped magenta nodes represent miRNAs, the triangle-shaped magenta nodes represent TFs, and the round rectangle-shaped red nodes represent the PD-specific genes

The other FFL is among the SPRK2 gene, hsa-miR-17-3p and SPI1. The TransmiR information indicated that hsa-miR-17-3p is inhibited by the SPI1 TF. By combing the TransmiR and DIANA-Tarbase data, we found that SPI1 and hsa-miR-17-3p both regulate the expression of its target gene SPRK2. Another study showed that miR-17 is upregulated in multiple system atrophy [71] and reduced during neurogenesis [72]. By combining this information, we elucidated possible relationships among the genes, miRNAs and TFs in FFLs in neurodegenerative disease by using Cytoscape software v. 3.4.0 (Fig. 8). Further study on these FFLs may help us understand the molecular biology of PD.

### Significance of IncRNAs and mTFs associated with these PD-specific miRNAs related to the PD-specific genes

The network showed the relationship among the PD-specific miRNAs, mTFs and lncRNAs. In this regulatory network, we identified 45 lncRNAs associated with these PD-specific miRNAs. The regulations of 31mTFs associated with 14 PD-specific miRNAs showed that most of these interactions had an activating effect (Table 6). The regulation of hsa-miR-204-3p of this network is noteworthy. It is associated with one lncRNAs but has no mTFs associated with it (Table 6). This miRNA represses HSPA6 which is known to be involved in PD [59]. In contrast to the above findings, hsa-miR-181a-5p has a maximum number of lncRNAs (15 lncRNAs) associated with it (Table 6). This miRNA is in turn repress one gene namely SRPK2. This gene is known to be involved in PD [34]. However, the miRNA is associated with only one mTFs (TP63) (Table 6). In order to find out the functional role of these 45 lncRNAs, we further analyzed them in EVLncRNAs database (http://biophy.dzu.edu.cn/EVLncRNAs/) [73]. Two lncRNAs (NEAT1 and TUG1) were found to be associated with neurodegenerative disease. Nuclear paraspeckle assembly transcript 1 (NEAT1) is known to be involved in neurological disorders including amyotrophic lateral sclerosis [74] and Huntington’s disease [75]. Taurine up-regulated 1 (TUG1) is known to be involved in neurological disorders including Huntington’s disease [76]. But their functions in PD were not identified by previous studies.Since these two lncRNAs of this regulatory network are associated with PD-specific miRNAs of our study, they might play an important role in PD.

### Significance of SNP analysis of the common PD-specific and other neurodegenerative disease-specific genes

Two SNPs were identified to be associated with the SNCA gene (shown in Table 8), which is in turn controlled by miRNAs including the PD-specific miRNA hsa-miR-153. The dbSNP database showed that SNCA is located on chromosome 4. SNPs rs17016074 and rs356165 are location in the SNCA gene, which is related to PD [77, 78]. Table 8 shows that these two SNPs are identified to be associated with SNCA gene which is in turn controlled by PD-specific miRNA hsa-miR-153 and this finding was experimentally validated [77, 78]. This strengthens the association of these two SNPs in PD. In addition, SNPs rs17016074 and rs356165 in SNCA gene associated with hsa-miR-7 is involved in PD and it was experimentally validated. However, hsa-miR-7 was not previously linked with PD. Therefore, hsa-miR-7 may be a significant PD epigenetic biomarker that requires further study.

We also analyzed SNPs associated with several neurodegenerative disease-specific genes that are in turn controlled by several miRNAs(shown in Table 9). Twenty-three SNPs were identified from four neurodegenerative disease-specific genes. HLA-C was found to be located on chromosome 6 and several previous studies have found the function of HLA-C in multiple sclerosis that was activated by killer cell immunoglobulin like receptor, two Ig domains and long cytoplasmic tail 2(KIR2DL2) [79]. The SNP rs1049853 in HLA-C was identified in multiple sclerosis patients and was controlled by the miRNA hsa-miR-4428. BCL2 is located on chromosome 18; SNP rs1016860 is located in the BCL2 gene, and the two possible nucleotide variations (A or G) are considered alleles for this base position. This SNP is regulated by several miRNAs: hsa-miR-27a-5p, hsa-miR-3127-5p, hsa-miR-3158-3p, hsa-miR-4720-3p, hsa-miR-4789-5p and hsa-miR-629-5p. MiR-27a was previously shown to be involved in neurodegenerative diseases, such as Huntington’s disease and Alzheimer disease [80, 81]. MiR-3127 and miR-629 were shown to play a role in cancer [82, 83]. CARHSP1 is located on chromosome 16 and there are four SNPs (rs1058929, rs9953, rs1058967 and rs2447924) at this specific base position; three possible nucleotide variations (A, G or T) are considered alleles for the rs1058929 base position, two possible nucleotide variations (G or T) are considered alleles for rs9953 and rs2447924 base positions, and two possible nucleotide variations (G or A) are considered alleles for the rs1058967 base position. These four SNPs may be regulated by several miRNAs: hsa-miR-1224-5p, hsa-miR-1265, hsa-miR-3605-5p, hsa-miR-3915, hsa-miR-4710, hsa-miR-5585-5p, hsa-miR-193a-5p, hsa-miR-3190-3p, hsa-miR-3615, hsa-miR-335-5p, hsa-miR-451b, hsa-miR-4533, hsa-miR-4797-5p, hsa-miR-3679-3p and hsa-miR-4286. MiR-1224 was found the fountion in PD targeting LRRK2 [84]. MiR-1265, miR-193a, miR-3615, miR-335 and miR-4286 were shown to play a role in cancer [85–88]. Two SNPs, rs6430498 and rs12512664, in miR-3679 were significantly associated with femoral neck bone mineral density [89]. Therefore, future studies should pursue the function of these SNPs and associated miRNAs in PD disease.

## Conclusion

In this study, we performed a meta-analysis with three microarray datasets from PD blood studies and five from PD SN studies to study DEGs, gene regulatory networks and lncRNA-mediated regulatory networks. The meta-analysis identified 36 common DEGs in PD blood studies and 17 common DEGs in PD SN studies. Further, we identified five PD-specific genes in our study: HSPA6, MAP2K6, SRPK2, NOL7 and SNCA genes. Analysis of the regulatory miRNAs associated with the common PD-specific genes resulted in the identification of PD-specific miRNAs (hsa-miR-204-3p, hsa-miR-17-3p, hsa-miR-181a-5p, hsa-miR-181b-5p, hsa-miR-181c-5p, hsa-miR-181d, hsa-miR-19a-3p, hsa-miR-19b-3p, hsa-miR-93-3p, hsa-miR-153, hsa-miR-23b-3p, hsa-miR-34a-5p, hsa-miR-9-5p and hsa-miR-7-5p). Analysis of the mTF-miRNA-gene-gTF network also led to the identification of two FFLs: one FFL between the SPRK2 gene, hsa-miR-19a-3p and SPI1 and the other between the SPRK2 gene, hsa-miR-17-3p and SPI. In the lncRNA-mediated regulatory network, 45 lncRNAs were associated with known PD-specific miRNAs in our study. Notably, these were not identified in previous studies and warrant further investigation as they might be important epigenetic regulators in PD. Moreover, SNP analysis identified two significant SNPs associated with PD-specific genes and six regulatory miRNAs, and seven significant SNPs associated with other neurodegenerative disease-specific genes and 23 regulatory miRNAs. These SNPs could be considered as latent risk factors for further validation. Thus, the findings of our study should be explored in further investigation of PD.

## Additional file

Additional file 1: **Table S1**. GO biological processes analysis of diferentially expressed genes in blood of Parkinson’s Disease. **Table S2**. GO biological processes analysis of diferentially expressed genes in substantia nigra of Parkinson’s Disease. **Table S3**. The PPI information for common DEGs in blood obtained from STRING online software. **Table S4**. The PPI information for common DEGs in substantia nigra obtained from STRING online software. **Table S5**. The mTF-miRNA-gene-gTF regulatory network of common PD-specific genes identified in blood datasets. **Table S6**. The mTF-miRNA-gene-gTF regulatory network of common PD-specific genes identified in brain substantia nigra. **Table S7**. The mTF-miRNA-gene-gTF regulatory network of common genes identified in blood datasets(other than PD-specific genes). **Table S8**. The mTF-miRNA-gene-gTF regulatory network of common genes identified in substantia nigra datasets(other than PD-specific genes). (XLS 1121 kb)

## Abbreviations

BP: Biological processes
DE: Differentially expressed
FFL: Feed forward loop
gTF: Gene transcription factor
HC: Healthy control
lncRNA: Long non-coding RNA
miRNA: microRNA
mTF: microRNA transcription factor
PD: Parkinson’s disease
PPI: Protein protein interaction
SN: Substantia nigra
SNP: Single nucleotide polymorphism
TF: Transcription factor
